# Nodulation by *Sinorhizobium meliloti* originated from a mining soil alleviates Cd toxicity and increases Cd-phytoextraction in *Medicago sativa* L.

**DOI:** 10.3389/fpls.2015.00863

**Published:** 2015-10-14

**Authors:** Tahar Ghnaya, Majda Mnassri, Rim Ghabriche, Mariem Wali, Charlotte Poschenrieder, Stanley Lutts, Chedly Abdelly

**Affiliations:** ^1^Laboratoire des Plantes Extremophiles, Centre de Biotechnologies de la Technopole de Borj CedriaHammam Lif, Tunisia; ^2^Departamento de Fisiologia Vegetal, Facultad de Ciencias, Universidad Autonoma de BarcelonaBarcelona, Spain; ^3^Groupe de Recherche en Physiologie Végétale, Earth and Life Institute – Agronomy, Université Catholique de LouvainLouvain-la-Neuve, Belgium

**Keywords:** phytoremediation, *Medicago sativa*, *Sinorhizobium meliloti*, cadmium, symbiotic association

## Abstract

Besides their role in nitrogen supply to the host plants as a result of symbiotic N fixation, the association between legumes and Rhizobium could be useful for the rehabilitation of metal-contaminated soils by phytoextraction. A major limitation presents the metal-sensitivity of the bacterial strains. The aim of this work was to explore the usefulness of *Sinorhizobium meliloti* originated from a mining site for Cd phytoextraction by *Medicago sativa*. Inoculated and non-inoculated plants were cultivated for 60 d on soils containing 50 and/or 100 mg Cd kg^−1^ soil. The inoculation hindered the occurrence of Cd- induced toxicity symptoms that appeared in the shoots of non-inoculated plants. This positive effect of *S. meliloti* colonization was accompanied by an increase in biomass production and improved nutrient acquisition comparatively to non-inoculated plants. Nodulation enhanced Cd absorption by the roots and Cd translocation to the shoots. The increase of plant biomass concomitantly with the increase of Cd shoot concentration in inoculated plants led to higher potential of Cd-phytoextraction in these plants. In the presence of 50 mg Cd kg^−1^ in the soil, the amounts of Cd extracted in the shoots were 58 and 178 μg plant^−1^ in non-inoculated and inoculated plants, respectively. This study demonstrates that this association *M. sativa*-*S. meliloti* may be an efficient biological system to extract Cd from contaminated soils.

## Introduction

During the last two decades, soil contamination with heavy metals, especially Cd became an increasing word problem in relation to its high toxicity for all living organisms. The major sources of Cd contamination result from anthropogenic activities, including industrial and mining processes (e.g., pigments, electroplating, batteries etc.), and Cd is a well known by-product of Zn smelting and steel factories. Moreover, Cd is frequently present in conventional (phosphate) and non-conventional fertilizers (sewage sludge, compost etc.). This makes it an ubiquitous dangerous pollutant that is now widespread in both urban and agricultural areas (He et al., 2005). Among the main heavy metals found in contaminated soils (Pb, Zn, Cu etc.), Cd is one of the most mobile, toxic, and carcinogenic element (Waalkes, 2003) and its presence in water resources and/or to the food chain thus poses a serious public health problem.

Being non-degradable, metal pollutants must be removed from contaminated soils. Several approaches including, physical, chemical, and biological methods have been tested to extract Cd pollutant from contaminated substrates. Unfortunately, due to their high costs, physicochemical procedures could not be applied in low-incomes countries. Moreover, these procedures are often destructive for soil microorganisms, leading to the inhibition of soil-biological activity and decrease in soil fertility. It is thus necessary to develop low-cost biological alternative methods which could be used to extract Cd from polluted soils and preserve or even increase soil biological properties. Phytoremediation is an emergent non-conventional approach based on the use of plants to cleanup or stabilize metal-polluted soils. It recently appeared as an efficient approach to clean-up disturbed environment. The major advantage of this remediation method compared to physicochemical technologies mainly results from its low cost, the possibility to apply it to a mix of several pollutants, and the fact that phytoremediation has no destructive effects on soil texture and microfauna.

The success of heavy-metal phytoextraction is a direct function of several parameters related to soil properties and considered plant species. Indeed, many plant species are not able to grow on polluted soils due to inappropriate physicochemical conditions as soil compaction, detrimental pH, nutritional deficiencies, and poor water availability (Wong, 2003). As a consequence, the ideal candidates for phytoextraction should not be only metal tolerant, but should also well adapted to other constraints. The identification of new species gathering the above mentioned characteristics is a challenging task for environmentalists and botanists.

Recent studies suggested that the symbiotic association between plants and microorganisms (bacteria and fungi) could enhance the capability of the host plants to accumulate and tolerate toxic metals within shoot tissues (Teng et al., 2015). It has been postulated that the association between plants and plant-growth-promoting rhizobacteria (PGPR) plays a significant role in enhancing metal availability and alleviating metal toxicity in several host plant species (Jayabarath et al., 2009; Cetin et al., 2011; Hao et al., 2014). Symbiotic microorganisms may increase the absorption and accumulation of metals but also detoxify them inside tissues through several mechanisms such as: (1) the efflux of metal ions outside the cytoplasm, (2) compartmentalization of metals inside the cell vacuoles (Antony et al., 2011), (3) biotransformation of toxic metal to less toxic forms (Cheung and Gu, 2007; Shukla et al., 2009), and (4) adsorption/desorption of metals especially on cell walls (Mamaril et al., 1997; Johnson et al., 2007).

Among the plant species establishing symbiosis with terrestrial bacteria, legumes are able to improve soil fertility and more especially enhance the N pool in the soil ecosystem through symbiotic N fixation. Hence, the establishment of symbiotic association with diazotrophs provides reduced nitrogen to the plant in exchange for carbohydrates (Kouchi et al., 2010). It has been shown that the inoculation of legumes with diazotrophic bacteria allows the host plants to improve growth under diverse stress conditions (Carrasco et al., 2005). Rhizobia can tolerate the high concentrations of metals present in soils polluted by mining and industrial activities (Mandal et al., 2008; Yang et al., 2009; Zribi et al., 2012). Moreover, owing to the strong relationship between micro and macro-partner of the symbiosis, the tolerance to toxic metals exhibited by the bacteria could modulate the response of its host plant to these pollutants. Although several works demonstrated that PGPR increase the tolerance of different plant species [e.g., *Pseudomonas tolaasi* ACC23/*Brassica napus* (Dell'Amico et al., 2008), *Flavobacterium* sp. 5P-4/*Brassica juncea* (Belimov et al., 2005), and *Burkholderia* sp. J62/maize and tomato (Jiang et al., 2008)], it would be of considerable interest to exploit the natural symbiotic association between legumes and rhizobia for rehabilitation of heavy metal contaminated soils by the phytoextraction process.

The aim of this study was thus to assess the usefulness of *Sinorhizobium meliloti* originating from a mining site for Cd phytoextraction by *Medicago sativa*. For this purpose, we investigated the effect of the symbiosis between the fast growing legume *M. sativa* and a bacterial strain purified from a metal-contaminated mining soil. The ability of the host plant to tolerate and accumulate Cd in the shoots was estimated in relation to growth on a Cd-contaminated soil.

## Materials and methods

### Soil sampling, characterization, and contamination

Loam-silt soil (20% clay, 58% silt, and 22% sand) was collected from the surface horizon (0–20 cm depth) in Borj Cedria region (Tunisia; 36° 44′ 33″ N 10° 19′ 22″ E). Its physicochemical properties were determined using classical methods and are shown in Table 1.

**Table 1:** **Physical and chemical characteristics of the soil used in the experiment**.

**Parameters**	**Values**
pH	7.6±1.52
Relative humidity (%)	1.2±0.08
EC (mmhos cm-1)	0.06±0.004
Organic carbon (g kg^−1^)	9.2±1.15
Organic matter (g kg^−1^)	17.5±2.05
Total nitrogen (g kg^−1^)	1±0.4
C/N	9.2±1.3
Phosphorus (mg kg^−1^)	8.2±1.17
K^+^(g kg^−1^)	0.38±0.02
Ca^2+^(mg kg^−1^)	0.26±0.05
Mg^2+^(g kg^−1^)	0.52±0.02
Na^+^(g kg^−1^)	1.31±0.045

*All values are the means of four replicates*.

The soil was spiked with CdCl_2_ to achieve two contamination levels of 50 and 100 mg Cd kg^−1^ soil. After adding Cd, the soil was equilibrated for 21 d during three cycles of saturation with tap water followed by air drying. Then, all soil samples (contaminated or not) were sterilized by autoclaving at 121°C for 1 h. Sterilized soils for the three treatments (0, 50, and 100 mg Cd kg^−1^ soil) were distributed in sterilized pots (2 kg of soil per pot). Each treatment consisted of 24 pots.

### Plant material and experimental design

Seeds of *M. sativa* L. cv Gabes were sown in each pot at a density of five seeds per pot (2 cm depth). After 1 week, seeds sown on contaminated and non-contaminated soils had germinated and seedlings had two first emerged leaves. One week later, subsequent clearing was performed to obtain the final plant density of two seedlings per pot. At this stage the inoculation with *S. meliloti* was performed. The *S. mliloti* strain had previously been purified from a mining soil containing 25 mg Cd kg^−1^ soil (Zribi et al., 2012). For each treatment, half of the pots were inoculated by adding 1 ml of a *S. meliloti* suspension containing 10^8^ ml^−1^ of bacteria close to the root system of each plant. During the culture period (60 d) under greenhouse conditions, plants were irrigated with controlled tap water free of organic or mineral pollution. Proper care and maintenance were afforded from the starting time of seed sowing to final plant harvest. At the final harvest, roots were carefully removed from the substrate, separated from the shoots, dipped in a cold solution of HCl (0.01 M) during 5 min to eliminate heavy metals adsorbed at the root surface (Aldrich et al., 2003), then washed three times with cold distilled water and blotted with filter paper. Shoots were then separated into leaves and stems. The fresh weight (FW) was measured immediately, and the dry weight (DW) after 48 h of desiccation in an oven at 60°C.

### Cations concentration

Dried samples (c.a. 100 mg) were ground to a fine powder using a pre-chilled porcelain mortar and pestle and digested in 4/1 (v/v) HNO_3_/HClO_4_ (20 ml) mixture at 100°C. After total evaporation, 30 ml of HNO_3_ 0.5% were added and Cd^2+^, Mg^2+^, Fe^2+^, and Ca^2+^ concentrations were determined by atomic absorption spectrometry (Spectra AA 220 FS). Potassium concentration was determined by flame spectrometry (Corning photometer).

The nitrogen content in dried tissues was determined by the Kjeldahl method. The dried samples were placed in digestion tubes and treated with the catalyst mixture (CuSO${}_{4}^{+}$K_2_SO${}_{4}^{+}$Se) and sulfuric acid. After a brief mixing, the digestion was initiated at 100°C and temperature was gradually increased to reach 350°C until complete digestion. After cooling to room temperature, the digestion solution was distilled with sodium hydroxide in order to convert the ammonium salt to ammonia which was then condensed in a conical flask with boric acid solution. The amount of ammonia present was determined by titration with sulfuric acid (N/100).

### Parameters used for analysis of Cd accumulation

#### Cd contents extracted in the shoots

The Cd content accumulated in the shoots of inoculated and non-inoculated plants was calculated as the product between Cd shoot concentrations by shoot biomass. Cd (μg plant^−1^) = Cd shoots (μg g^−1^ DW)^*^ shoot biomass (g plant^−1^).

#### Bioconcentration factor

The Cd^2+^ uptake, was depicted by a bioconcentration factor (BCF), which provides an index of the plant's ability to accumulate metal with respect to the concentration of this pollutant in the soil. It is calculated as follows:

BCF = Cd^2+^ concentration in dry shoots (μg g^−1^)/Initial Cd concentration in dry soil (μg g^−1^).

#### Translocation factor

The translocation factor (TF) depicts the ability of the species to translocate the metal from the roots to the shoots.

TF = Cd^2+^ in dry shoots (μg g^−1^)/Cd^2+^ in dry roots (μg g^−1^).

### Statistical analysis

Analyses of variance (ANOVA) with orthogonal contrasts and mean comparison procedures were used to detect differences between treatments. Mean separation procedures were conducted using the multiple range tests with Fisher's least significant difference (LSD) (*P* < 0.05).

## Results

### Plant morphology and growth parameters

Seed germination was not affected by the 50 mg Cd kg^−1^ soil treatment, while an 8% reduction of the germination capacity was observed in response to 100 mg Cd kg^−1^ treatment. However, such a decrease was statistically non-significant as compared to the germination rates in non-contaminated soil.

**Figure 1:**
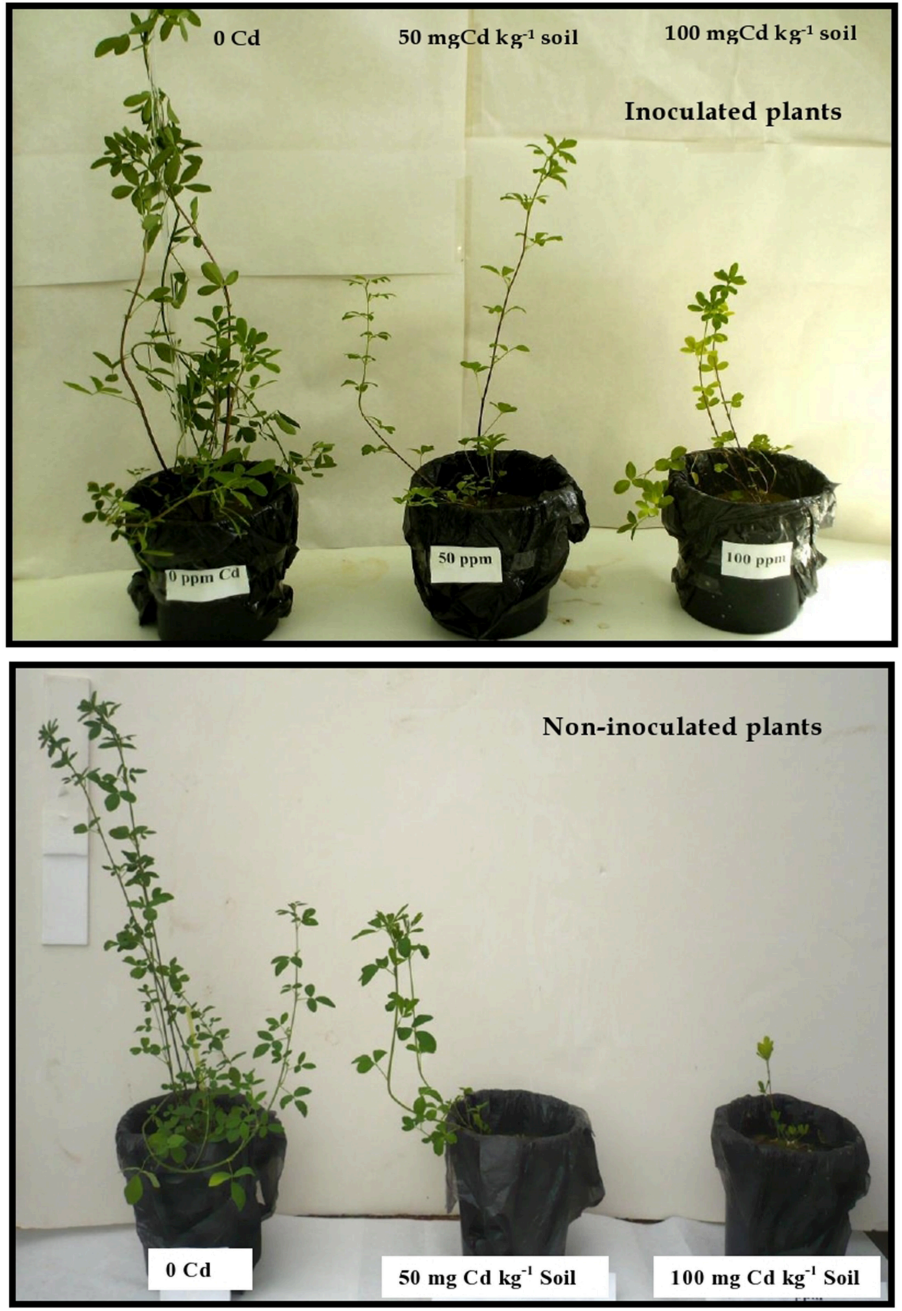
**Effects of the inoculation with ***Sinorhizobium meliloti*** on the morphology of ***Medicago sativa*** plants**. Inoculated and non-inoculated plants were cultivated during 60 d on soil containing 0, 50, or 100 mg Cd kg^−1^ soil.

The effect of different Cd soil-concentrations on the morphology of inoculated and non-inoculated plant 60 d after germination is shown in Figure 1. In the absence of Cd, inoculated plants exhibited a better development than non-inoculated ones. Cd supply significantly reduced plant growth and this deleterious effect was proportional to soil Cd concentration. Cadmium induced visual toxicity symptoms in the form of chlorosis and necrosis. These symptoms were more sever in the non-inoculated *M. sativa* plants (especially at 100 mg kg^−1^ of Cd) than in the inoculated ones.

**Figure 2:**
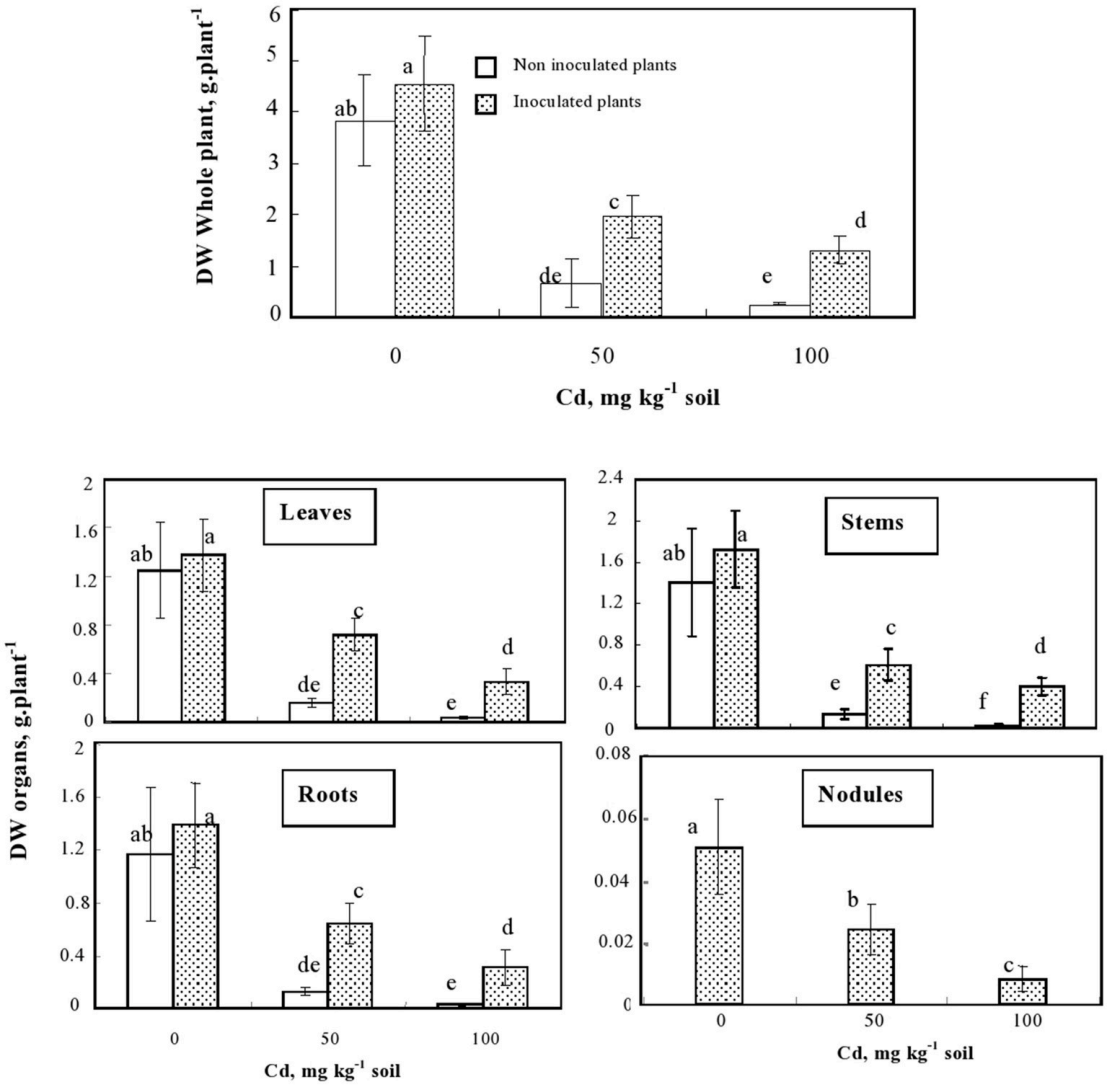
**Effects of the inoculation on the biomass (g plant^−1^) of whole plants and different plant organs of ***M. sativa*** cultivated during 60 d on soil containing different Cd doses**. Values are means ± SD (*n* = 12); values marked by different letters are statistically different (*p* < 0.05).

**Figure 3:**
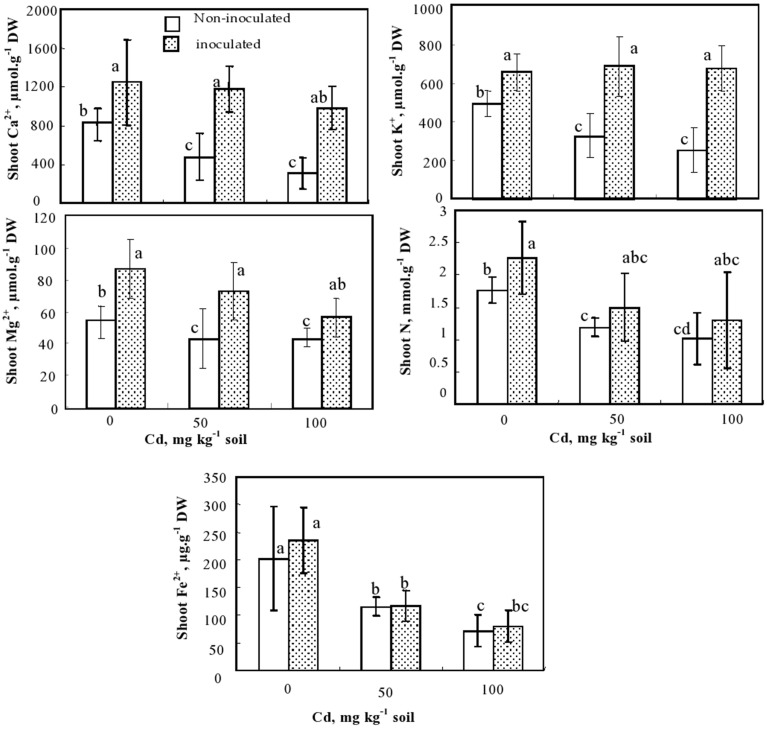
**Calcium, potassium, magnesium (μmol g^−1^ DW), nitrogen (mmol g^−1^) and Fe^2+^ (μg g^−1^ DW) concentrations in shoots of ***S***. ***meliloti*** inoculated and non-inoculated ***M***. ***sativa*** plants and cultivated during 60 d on soil containing different Cd doses**. Values are means ± SD (*n* = 8); values marked by different letters are statistically different (*p* < 0.05).

Inoculation of plants with the rhizobacteria enhanced the dry matter production by about 20% both at the whole plant level and in a similar way for each organ individually considered (Figures 2, 3). In the presence of Cd, the difference in DW between inoculated and non-inoculated *M. sativa* was even higher. Inoculated plants produced 2.5 and 5 fold more biomass than non-inoculated ones when cultivated in the presence of 50 and 100 mg Cd kg^−1^, respectively. Inoculation with *S. meliloti* reduced the decrease of biomass production caused by 50 mg Cd kg^−1^ in the soil from 82 to 60%.

Our results also confirmed that soil sterilization step prior to inoculation was successful since non-inoculated plants never developed root nodules. Regarding the effect of Cd on the nodulation process, we showed that the presence of the metal in soil reduced the number and the total biomass of nodules per plant (Figure 2). However, even at 100 mg Cd kg^−1^ soil, the nodules still exhibited a pinkish color, suggesting that they remained able to fix nitrogen. This demonstrates that the used strain of micro-symbiont (*S. meliloti*) was Cd-tolerant and able to cope with the considered Cd doses.

### Inoculation effect on *M. sativa* plants nutrition under Cd stress

The effect of Cd on nutrient (Ca, K, Mg, N, and Fe) concentrations in the shoots of inoculated and non-inoculated *M. sativa* plants is shown in Figure 3. Under control conditions and in the presence of the two tested Cd doses, *S. meliloti* inoculated plants accumulated more macronutrients Ca, K, Mg, and N than non-inoculated ones (Figure 3). Cadmium supply significantly reduced Ca, K, Mg, and N concentrations in the shoots of non-inoculated plants but had no impact on these nutrients in inoculated ones (Figure 3). Cadmium reduced shoot Fe concentrations in all plants (Figure 3).

### *S. meliloti* inoculation effect on Cd accumulation in *M. sativa* plants

**Figure 4:**
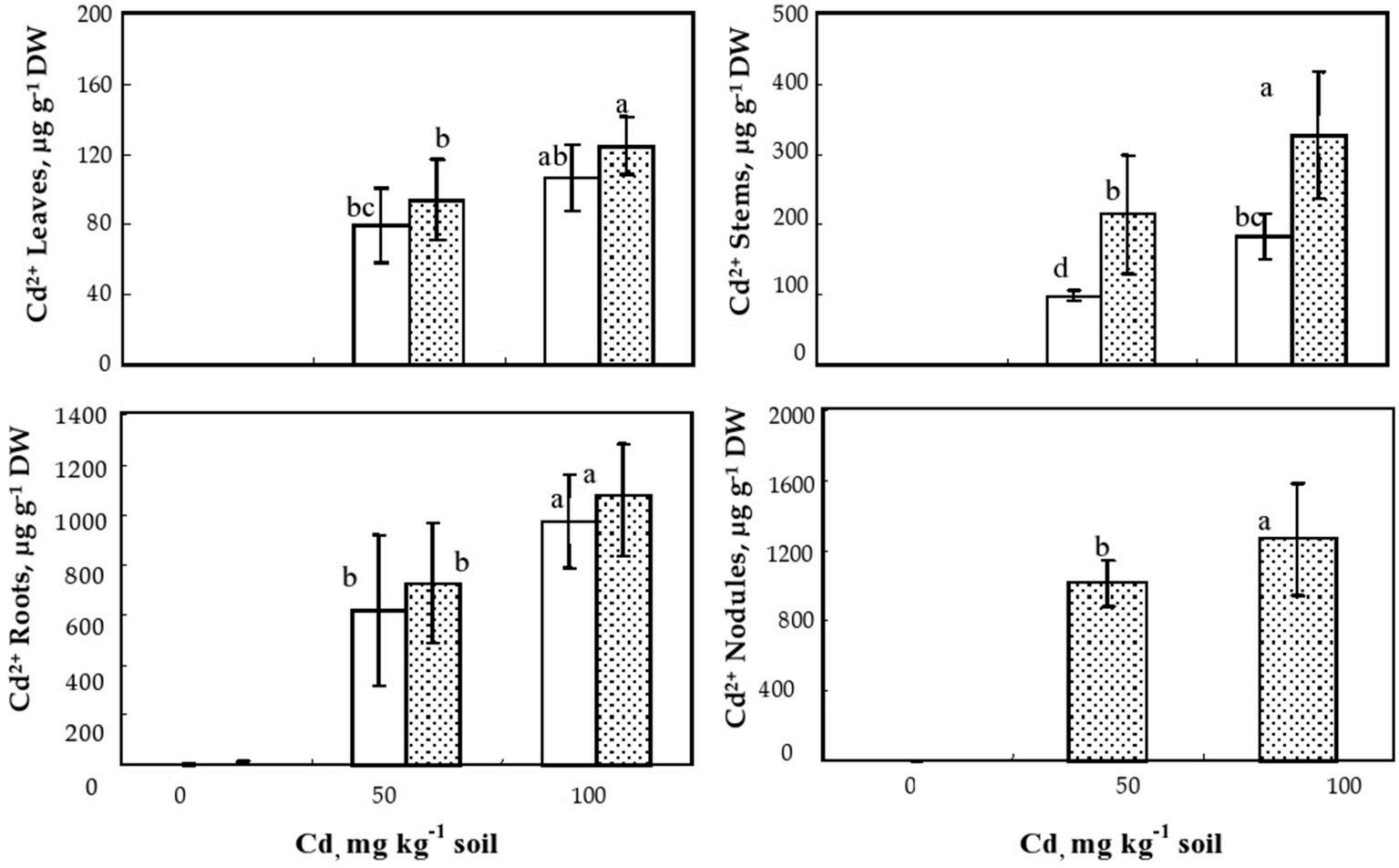
**Cadmium concentrations (μg g^−1^ dry weight) in leaves, stems, roots, and nodules, of ***Medicago sativa*** plants inoculated or not with ***Sinorhizobium meliloti*** and cultivated during 60 d on soil added with different Cd doses**. Values are means ± SD (*n* = 12); values marked by different letters are statistically different (*p* < 0.05).

Cadmium concentrations in the different organs of inoculated and non-inoculated *M. sativa* plants are given in Figure 4. As expected, Cd was undetectable in control plants but obviously increased with increasing Cd doses. The highest Cd concentrations were observed in nodules followed by roots and stems; the lowest Cd concentrations were always observed in the leaves (Figure 4). For both soil Cd levels, the inoculated plants accumulated higher Cd concentrations than non-inoculated ones. Nevertheless, this difference was statistically significant only for stems where the inoculated plant accumulated two fold higher Cd concentrations than non-inoculated plants (Figure 4).

**Figure 5:**
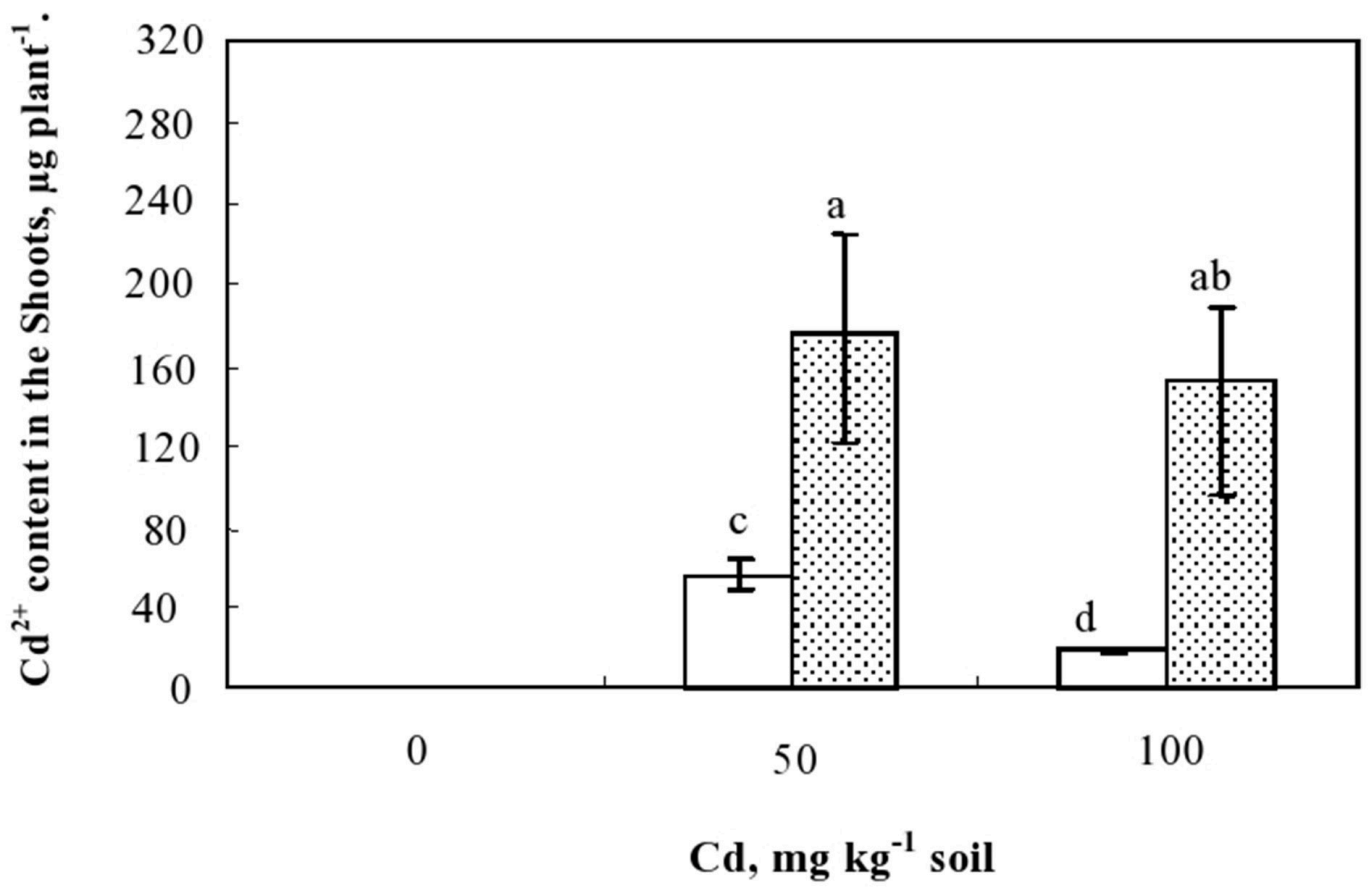
**Variation of Cd contents (μg plant^−1^) accumulated in the shoots of plants cultivated during 60 d in the presence of different Cd doses in the soil and inoculated or not with ***Sinorhizobium meliloti*****. Values are means ± SD (*n* = 12); values marked by different letters are statistically different (*p* < 0.05).

The potential of studied plants for Cd phytoextraction was further evaluated based on the total amounts of the metal accumulated in the shoots. This parameter, which represents the product of shoot biomass and their Cd concentrations, showed that inoculated plants extracted significantly more Cd than non-inoculated ones (Figure 5). Hence, in the presence of 50 mg kg^−1^ soil of Cd in the soil, the amounts of Cd extracted from the soil and translocated to the shoots were 58 and 178 μg plant^−1^ in non-inoculated and inoculated plants, respectively. This higher potential of Cd extraction by inoculated plants was mainly related to improved growth occurring concomitantly with the higher Cd-shoot concentration. The lowest Cd amount extracted by plant was detected in the non-inoculated plants cultivated in the presence of 100 mg Cd kg^−1^ soil and appeared as a consequence of the strong growth inhibition suffered by the plants under this extremely high Cd soil concentration.

In order to highlight the effect of inoculation on the capability of plants to absorb and to translocate Cd from the roots to the shoots, we determined both the bioconcentration (BCF) and the translocation (TF) factors (Table 2). Our data revealed that the inoculation significantly enhanced the aptitude of *M. sativa* to transport Cd through the xylem from the roots to the shoots. The values of BCF, which represents the ratio between Cd concentration in the shoots and Cd concentration in the soil, were higher than the unit suggesting that all plants cultivated on contaminated soils were able to concentrate the metal in their aerial parts. The symbiotic association *M. sativa-S. melilotti*, increased the BCF values up to three times. Taken together, our data indicate that the inoculation enhanced the capability of *M. sativa* to absorb and to transport Cd from the roots toward the shoots.

**Table 2:** **Variation of the Translocation factor [TF = Cd${}_{\text{shoots}}^{2+}$ (μg g^−1^)/Cd${}_{\text{roots}}^{2+}$ (μg g^−1^)] and Bioconcentration factor [BCF = Cd${}_{\text{shoots}}^{2+}$ (mg kg^−1^)/Cd_***soil***_ (mg kg^−1^)] values in inoculated and non-inoculated plants of ***M. sativa*** after 60 d of cultivation on soil contaminated with 50 and 100 mg kg^−1^ of Cd**.

**Cd_*soil*_ (mg kg^−1^)**	**Inoculated plants**		**Non-inoculated plants**	
	**50**	**100**	**50**	**100**
TF	0.49 ± 0.2 a	0.36 ± 0.1ab	0.12 ± 0.04d	0.23 ± 0.1c
BCF	4.3 ± 0.5 a	3.5 ± 0.9 a	1.88 ± 0.4 b	1.7 ± 0.1 b

*Values are means ± SD (n = 12); values followed by different letters in the same row are statistically different (p < 0.05)*.

## Discussion

The plant response to toxic metals in terms of tolerance and accumulation depends on several factors related to the species and environmental conditions including the physicochemical and biological characteristics of the soil. The modification of one or several parameters could impact the plant's capacity to tolerate and accumulate pollutants. One of the most studied systems tested in order to increase the plant resistance to metal constraint is the symbiotic association between plants and rhizospheric micro-organisms such as bacteria and fungi (Kidd et al., 2009). Legume–Rhizobium associations have been recently considered as promising biological systems to increase the capacity of host plants to tolerate and accumulate toxic heavy metals (Santi and Bhattacharyya, 2012; Hao et al., 2014). *M. sativa* is considered as an excellent fodder plant, due to its high biomass production and its relatively high tolerance to environmental stress factors, such as salinity and drought (Wang et al., 2011; Bertrand et al., 2015). It has also been recommended as a promising material for metal phytoextraction purposes (Wang et al., 2011; Zribi et al., 2012).

Our results showed that Cd did not significantly affect the germination capacity of *M. sativa* and suggest that *M. sativa* is tolerant to Cd at the germination stage, which justifies our investigation on Cd accumulation potential at subsequent vegetative stages. When considering the micro-symbiont associated to this plant, the works of Zribi et al. (2012) and Fan et al. (2011) clearly demonstrated that the bacteria (*S. meliloti*) nodulating alfalfa is able to survive in mining soils contaminated with various metals as Cd, Zn, Pb, and Cu. In the present work, we confirmed these finding since the *Sinorhizobium* strain originally isolated from a mining soil containing 25 mg Cd kg^−1^ soil remained able to nodulate *M. sativa* even in the presence of 100 mg Cd kg^−1^ in soil and still presented a pink color typical of functional root nodules.

Most physiological and biochemical processes controlling plant growth and development and operating after germination, such as photosynthesis and nutrition, are sensitive to Cd. Several authors demonstrated that plants growing in spiked soils contaminated with heavy metals for experimental purposes or in soils already contaminated exhibit severe toxicity symptoms. The deleterious effects of Cd consist in chlorosis and growth reduction (Mariem et al., 2014), mineral and photosynthesis disturbances and several morphological abnormalities (Amari et al., 2014; Taamalli et al., 2014). The mechanisms behind these toxic effects include Cd-induced inhibition of uptake and translocation of essential nutrients, especially Fe, Mg, and Zn, Cd-induced production of reactive oxygen species, and interference with essential enzymatic systems as well as hormonal imbalances (Barceló et al., 1986; Di Toppi and Gabbrielli, 1999; Rodríguez-Serrano et al., 2009).

In our study, we showed that inoculated and non-inoculated plants suffered from an acute chlorosis and growth reduction induced by Cd. However, these effects were more intense in non-inoculated plants suggesting that inoculation with *S. meliloti* alleviated Cd-induced toxicity in *M. sativa*. This improved plant performance was not due to a decrease of Cd uptake (Figure 2, Table 2), but to a better detoxification of Cd within tissues. Nodules are characterized by high levels of antioxidant defenses including nicotinamine, phytochelatins, and metallothioneins (Becana et al., 2010), which may contribute to detoxify Cd. Nodulation is a nutrient demanding process. Especially high concentrations of Fe, Mg, Ca, and K are required and these elements thus commonly accumulate in nodules (Brear et al., 2013). Nonetheless, in our study, shoot concentrations of these essential nutrients (Ca, Mg, K) increased or were maintained equal (Fe) to those recorded in non-inoculated plants. This behavior of plants inoculated with the beneficial *S. meliloti* contrasts with the data reported for Cd-treated plants infected by the pathogenic fungus *Botrytis cinerea* where a strong inhibition of Fe translocation from roots to shoots has been observed (Cabot et al., 2013). Infection of plants with diazotroph bacteria apparently implies up-regulation of nutrient uptake and transport. The ability to maintain higher Mg, Ca, and K shoot concentrations in the inoculated plants (Figure 3) may contribute to their improved performance under Cd stress. In addition, due to the symbiotic nitrogen fixation (SNF), the shoots of inoculated plants were more, but not significantly, concentrated in total N. This phenomenon could also help inoculated *M. sativa* to cope with internal Cd, due to the crucial role of N in the biosynthesis of Cd chelators in plants (Anjum et al., 2015).

The impact of inoculation with micro-organisms on host plant-responses to heavy metals are poorly documented, especially in the case of legumes plants and Cd stress. Nevertheless, other combinations of host plant-microbes have been shown to increase plant resistance to heavy metals. For example, Ahemad and Khan (2010) demonstrated the positive effects of *Mesorhizobium* sp. on chickpea yield under different metal-stress induced by addition of herbicides. Wani et al. (2009) and Wani and Khan (2010) demonstrated respectively that *Bacillus* sp. PSB10 and *Mesorhizobium* sp. RC3 ameliorated the growth and alleviated phytotoxicity in *Cicer arietinum* cultivated under Cr stress. In *Glycine max*, the inoculation with *Bradyrhizobium japonicum* Cb1809 alleviated As-induced growth reduction. Moreover, *Bradyrhizobium japonicum* has recently been shown to improve Cd tolerance not only of its host plant but also of non-leguminous species (Reichman, 2014). The mechanisms leading to the reduction of metal toxicity in host plants induced by the microsymbiont are essentially related to metal sequestration and detoxification. This effect could start with the restriction of metal uptake by the root cell of host plant as a result of their accumulation by the bacteroids inside the nodules (Ike et al., 2007). In plant tissues, it has been suggested that the inoculation is also able to enhance the fixation of excess of metal on cell wall and their compartmentalization into vacuoles, thus avoiding their toxic accumulation into the cytosol (Antony et al., 2011; Cetin et al., 2011; Sofia et al., 2012). These effects could be responsible for the restoration of several biochemical processes in inoculated plants as compared to non-inoculated ones when subjected to toxic levels of metals. In the same context, Gupta et al. (2007), Ganesan (2008), and Wani and Khan (2010) showed that inoculation with N-fixing bacteria enhanced chlorophyll biosynthesis, protein content and vital enzymes activities in various plant species subjected to different toxic metals. Wani et al. (2007) reported that the inoculation of green gram plants (*Vigna radiata* L.) with PGP *Bradyrhizobium* sp. RM8 tolerant to Ni and Zn enhanced leghemoglobin content, seed yield, and grain protein concentration in plants growing on a soil contaminated with 290 mg Ni kg^−1^ and 4.89 g Zn kg^−1^. The authors suggested that the increased plant growth and the improvement of biochemical processes induced by *Bradyrhizobium* sp. under metal constraint was possibly due to the effect of bacterial metabolites such as phytohormones, siderophores, and ammonia (Wani et al., 2007). More recently, the same authors (Wani et al., 2008) found that inoculation of pea (*Pisum sativum*) grown in metal-contaminated soil (580 mg Ni kg^−1^, 9.58 g Zn kg^−1^) with metal-tolerant *Rhizobium* sp. RP5 increased plant biomass by 46–65% in the presence of Ni and by 47–54% when Zn was added.

Quantification of shoot Cd concentration lead us to conclude that, concomitantly to the alleviation of Cd toxicity, inoculation also significantly enhanced Cd accumulation in inoculated plants as compared to non-inoculated one (Figure 4, Table 2). We suggest that the improvement of Cd resistance induced by *S. meliloti* in *M. sativa* was not due to an exclusion process but resulted from detoxification mechanisms. Cadmium translocation from the root to the shoot was clearly enhanced by inoculation with *S. meliloti* in alfalfa (Figure 5, Table 2). The increase of metal uptake in inoculated plants could be induced by the enhancement of metal bioavailability in soil by the bacteria. Indeed, it well known that the free-living or plant symbiotic soil micro-organisms could reduce the interactions between bivalent cations and soil components. This property may be responsible for the reduction of metal adsorption and the enhancement of heavy metal release in soil solution (Kurek and Majewska, 2012), especially in Zn rich soils. In our study, the increase of soil Cd mobility induced by *S. meliloti* might explain the increase of the bioconcentration factor. In contrast, Wani et al. (2007, 2008) showed that the symbiotic associations *Bradyrhizobium* sp*/Vigna radiata* L. and *Rhizobium* sp /*Pisum sativum* induced reduction of the metal concentrations in plant organs of inoculated plants as compared to non-inoculated ones in the presence of elevated concentration of Zn and Ni.

Taken together, our daa suggest that the symbiotic association *Sinorhizobium. Meliloti*/*M. sativa* could be an efficient tool for the rehabilitation of Cd contaminated soils by phtoextraction. The selected strain of microsymbiont is indeed tolerant to various heavy metals as Cd, Pb, and Zn (Zribi et al., 2012) and it improves plant growth, Cd absorption and translocation to the shoots of *M. sativa*.

## Conclusion

Considering the mineral soil composition used in this study, the symbiotic association between the host plant *M. sativa* and the used bacterial strain of *S. meliloti* constitutes an efficient biological combination to extract Cd from contaminated soils. Besides the normal elevated biomass production shown by this species under different abiotic constraints when living in symbiotic association with *S. meliloti*, this inoculation also alleviated the toxic effects of Cd on plant morphology, growth and mineral nutrition. It increases the total amount of Cd extracted from the soil and accumulated in the shoot as a result of the enhancement of Cd absorption (bioconcentration factor) and translocation (translocation factor) processes occurring concomitantly with the improvement of plant growth under Cd stress. Owing to its high biomass production, its relative tolerance to Cd and its high potential to accumulate Cd in the shoots when inoculated with an appropriate strain of *S. meliloti, M. sativa* could be considered as promising candidate for Cd phytoextraction from contaminated soils. However, this potential might be impacted by high Zn levels in the soils.

### Conflict of interest statement

The authors declare that the research was conducted in the absence of any commercial or financial relationships that could be construed as a potential conflict of interest.
